# ^89^Zr-Radiolabeled Trastuzumab Imaging in Orthotopic and Metastatic Breast Tumors

**DOI:** 10.3390/ph5010079

**Published:** 2012-01-05

**Authors:** Albert J. Chang, Ravindra DeSilva, Sandeep Jain, Kimberley Lears, Buck Rogers, Suzanne Lapi

**Affiliations:** 1 Department of Radiation Oncology, Mallinckrodt Institute of Radiology, Washington University School of Medicine, St. Louis, MO 63110, USA; Email: achang@radonc.wustl.edu (A.J.C.); klears@radonc.wustl.edu (K.L.); brogers@radonc.wustl.edu (B.R.); 2 Department of Radiological Sciences, Mallinckrodt Institute of Radiology, Washington University School of Medicine, St. Louis, MO 63110, USA; Email: deslivar@mir.wustl.edu (R.D.); Sandeep.Jain@sparcmail.com (S.J.)

**Keywords:** HER2, PET, ^89^Zr, immunoPET

## Abstract

The human epidermal growth factor receptor 2 (HER2/neu) is overexpressed in 20–30% of breast cancers and is associated with tumor growth, angiogenesis, and development of distant metastases. Trastuzumab, an anti-HER2 monoclonal antibody, is used for the treatment of HER2 positive breast cancer and clinical efficacy of this agent is dependent on HER2 expression. Targeted PET imaging of HER2 with radiolabeled trastuzumab may be used to determine HER2 expression levels and guide therapy selection. The purpose of the current study was to evaluate a facile ^89^Zr-trastuzumab preparation method that can be efficiently applied for clinical grade production. Also, relative HER2 expression levels in orthotopic and metastatic breast cancer models were assessed by PET imaging using the ^89^Zr-trastuzumab produced by this simpler method.

## 1. Introduction

The human epidermal growth factor receptor 2 (HER2) is a member of the epidermal growth factor receptor (EGFR) tyrosine kinase family [1]. Heterodimerization of HER2 with other members of the EGFR family promotes cell proliferation, survival, and migration. HER2 gene amplification and overexpression of HER2 protein is present in multiple malignancies including 20–30% of invasive breast cancers [2,3,4,5]. The association of HER2 with resistance to hormone therapy, chemotherapy, and conventional radiation therapy makes it an attractive target for breast cancer treatment [2,3,6].

Trastuzumab (Herceptin^TM^, Genentech, South San Francisco, CA, USA) is a monoclonal antibody that targets the extracellular domain of HER2 and is widely used for the treatment of HER2 positive breast cancer [7]. The standard of care for locally advanced HER2 positive breast cancer is combination treatment with trastuzumab, chemotherapy, and radiation therapy [8]. In addition to use as a first-line therapy, trastuzumab is used in the recurrent and metastatic settings [7]. An improvement in survival was seen in patients with HER2 positive metastatic disease receiving trastuzumab [9,10].

Although multiple studies have confirmed that trastuzumab is beneficial for the treatment of HER2 positive breast cancer [3,7,9,10,11], the clinical efficacy is dependent on the level of HER2 expression [12]. Two FDA-approved techniques exist to evaluate HER2 expression level, including immunohistochemistry (IHC) and fluorescence *in situ* hybridization (FISH) [13]. Discrepancies have been observed between the two methods [14]. In addition, HER2 expression can vary over the course of therapy [15,16] and over the course of the disease [15]. HER2 expression has been demonstrated to be discordant between the primary lesion and distant metastatic lesions, and, moreover, may vary across metastatic lesions [17,18,19,20]. Thus, the use of repeated biopsies during the course of the disease may not be clinically feasible due to their invasiveness and not all lesions are readily accessible to be biopsied [21]. Therefore, a method that can reliably detect HER2 expression in individual lesions would be of critical importance in identifying patients who benefit from HER2-targeted therapy. Molecular imaging is a potentially less invasive solution for HER2 determination [22,23,24,25,26].

The development of radiolabeled antibodies and antibody fragments for cell-surface receptor detection is an active area of research [27]. Trastuzumab has been radiolabeled with ^111^In, ^64^Cu, and ^89^Zr, for *in vivo* SPECT and PET imaging of HER2 in xenograft models of ovarian and breast cancer [22,23,24,25]. ^89^Zr is an ideal radionuclide for evaluation of intact antibodies because the extended half-life of ^89^Zr (74.8 h) allows for imaging at 72–120 h when antibodies begin to equilibrate in the body [28].

Multiple studies have evaluated the use of ^89^Zr for studying antibody biodistribution [28,29,30,31,32]. Dijkers *et al*. studied the biodistribution of ^89^Zr-radiolabeled trastuzumab in 14 patients with HER2+ metastatic breast cancer [28] and demonstrated the feasibility of ^89^Zr-trastuzumab to detect HER2 expression *in vivo*. They utilized a multi-step procedure with a succinylated derivative of the bifunctional chelator, desferrioxamine B (*N*-sucDf), for conjugation to trastuzumab and subsequent radiolabeling with ^89^Zr [33]. Recently, a simpler method of chelator-antibody conjugation chemistry was described with the *p*-isothiocyanatobenzyl derivative of desferrioxamine B (Df-Bz-NCS) [34,35]. This two-step production method may be readily applied to good manufacturing production of ^89^Zr-trastuzumab. The purpose of the current study was to evaluate the facile preparation method for ^89^Zr-trastuzumab production and the utility of ^89^Zr-trastuzumab, as prepared by this two-step production method, to characterize HER2 expression in orthotopic and metastatic breast cancer models.

## 2. Experimental Section

### 2.1. Flow Cytometry

MDA435/LCC6^HER2/GFP/Luc^ (HER2+) and MDA435/LCC6^Vector^ (HER2−) cell lines, which overexpress and minimally express HER2/neu, respectively, were generous gifts from Dawn Waterhouse (BC Cancer Agency, Vancouver, BC, Canada). The HER2+ cell line constitutively expresses luciferase allowing for bioluminescent imaging in the presence of luciferin. Cells were grown in Dulbecco’s Modified Eagle Media supplemented with 10% fetal bovine serum until 80% confluent. Cells were harvested with Cell Dissociation Buffer (Invitrogen, Carlsbad, CA, USA), washed with PBS, and incubated on ice with 20 μg/mL trastuzumab. After 1 h, cells were washed with PBS and incubated with Alexa Fluor 546 goat anti-human IgG (Invitrogen) for 30 min. HER2 expression was analyzed (30,000 events collected) with the FACSAria Flow Cytometer (Becton Dickinson, Franklin Lakes, NJ, USA).

### 2.2. ^89^Zr Production and Antibody Labeling

^89^Zr-oxalate was produced via the ^89^Y(p,n)^89^Zr transmutation reaction on the CS-15 cyclotron (Cyclotron Corporation, Berkeley, CA, USA) as described previously [33,36]. The resulting ^89^Zr-oxalate was produced with a specific-activity of 8.1–15.4 GBq/μmol (220–418 mCi/μmol). Trastuzumab was incubated with Df-Bz-NCS (Macrocyclics, Dallas, TX, USA) in 0.1 M NaHCO_3_ buffer pH 9.0 at room temperature for 30 min in a 1:5 molar ratio. The resulting product, Df-Bz-NCS-trastuzumab, was purified via Zeba Spin Desalting Columns (Pierce Biotechnology, Rockford, IL, USA). ^89^Zr was complexed with Df-Bz-NCS-trastuzumab at a ratio of 0.22 MBq/μg (6 μCi/μg) of antibody in 0.5 M HEPES buffer pH 7.0 at 37 °C for 1 h with constant agitation on an orbital mixer. Approximately 100 µg of antibody was used per reaction. ^89^Zr-trastuzumab was purified with Zeba Spin Desalting Columns and radiochemical purity was determined by radio-TLC using a mobile phase of 50 mM DTPA and analytical size-exclusion chromatography (Superose 12 10/300 GL, GE Healthcare, Piscataway, NJ, USA) with 20 mM HEPES and 150 mM NaCl (pH 7.3) eluted at a flow rate of 0.75 mL/min. Millenium 32 software (Waters, Milford, MA, USA) was used to quantify chromatograms by integration.

### 2.3. Immunoreactive Fraction

The immunoreactive fraction of ^89^Zr-trastuzumab was determined using a cell-binding assay, as previously described by Lindmo *et al*. [37]. HER2+ cells were suspended in microfuge tubes at increasing concentrations ranging from 0.5 to 5 × 10^6^ cells in 500 μL PBS. Approximately 37 kBq/0.25 μg of ^89^Zr-trastuzumab in 50 μL were added to each tube (n = 3) and agitated on an orbital mixer for 60 minutes at 25 °C. Cells were pelleted by centrifugation, washed twice with PBS, and subsequently counted for ^89^Zr activity. The specific binding was calculated as the ratio of bound radioactivity to the total amount of administered activity and was background corrected. To determine binding specificity, similar studies were performed with the addition of 100 μg of non-radiolabeled trastuzumab in HER2+ cells and also in HER2− cells.

### 2.4. Animal Studies

4 × 10^6^ HER2+ or HER2− cells were injected into the mammary fat pad of athymic nude mice 6–8 weeks of age. Tumors grew for 6 weeks until they reached approximately 10 mm in greatest dimension. A metastatic model was created by injection of 2 × 10^6^ HER2+ cells into the tail vein of athymic nude mice as previously reported [38]. As the HER2+ cells constitutively express luciferase, bioluminescent imaging was performed 6 weeks after tail vein injection to evaluate the development of metastatic lesions.

#### 2.4.1. Biodistribution Studies

*In vivo* biodistribution studies were performed to determine the uptake of ^89^Zr-trastuzumab in HER2+ and HER2− tumors in relation to normal organs. 0.55 MBq (15 μCi)/3.75 μg of ^89^Zr-trastuzumab was administered via intravenous tail vein injection. Mice were sacrificed at 24 and 96 h post-injection by cervical dislocation and tumor and select organs were harvested. Specific uptake for each tissue was measured with background and decay correction and expressed as % injected dose per gram of tissue (% ID/g) as calculated by normalization to the total activity injected.

#### 2.4.2. MicroPET/CT Studies

MicroPET/CT experiments were performed with the Inveon MicroPET/CT scanner (Siemens, Knoxville, TN, USA). Mice were administered ^89^Zr-trastuzumab(3.0–3.7 MBq (80–100 μCi)/20–25 μg in 100 μL 0.9% sterile saline) via tail vein injection when orthotopic tumors reached 10–15 mm in greatest dimension or when bioluminescent areas were observed on the BLI scans for the metastatic model. At 24 and 96 h post-injection, mice were anesthetized with 2% isoflurane and imaged. Static images were collected for 20 min and co-registered with image display software (Inveon Research Workplace Workstation, Siemens, Schenectady, NY, USA). Regions of interest were drawn and the mean standard uptake values for tumors were determined using the formula: SUV = [(MBq/mL) × (animal wt. (g))/injected dose (MBq)].

### 2.5. Immunohistochemistry

Tumors were harvested after completion of MicroPET/CT imaging studies and immediately fixed in 10% formalin. After allowing for the radioactivity to decay to background levels, tumors were embedded in paraffin. Five-micron sections were prepared and blocked in Dako Protein Block (Dako, Carpinteria, CA, USA) for 30 min at room temperature. Antigen retrieval was performed in citrate-based buffer using a pressure cooker (Biocare Medical). The sections were incubated with rabbit polyclonal anti-HER2 primary antibody (1:200, Abcam, Cambridge, MA, USA) overnight at 4 °C and visualized with Alexa Fluor 555-conjugated goat anti-rabbit IgG (1:200, Invitrogen). Sections were mounted were mounted with SlowFade Gold antifade reagent with 4,6-diamidino-2-phenylindole (DAPI) (Invitrogen) and cover slipped. Images were obtained with a Soft Imaging Solutions FVII cooled monochrome digital camera, Peltier cooled to −10 °C (Olympus America, Center Valley, PA, USA).

### 2.6. Statistical Analysis

The unpaired, two-tailed Student’s *t*-test was utilized for data analysis. Differences at the 95% confidence level (P < 0.05) were considered to be statistically significant.

## 3. Results and Discussion

### 3.1. Trastuzumab Conjugation, Radiolabeling, and Stability Testing

Trastuzumab was successfully conjugated to Df-Bz-NCS and radiolabeled with ^89^Zr. The radiolabeling efficiency was 78.4% ± 11.3%, the radiochemical purity was 98.7 ± 1.2%, and the specific activity was 136.9 ± 22.2 MBq/mg (3.7 ± 0.6 mCi/mg) (n = 9).

### 3.2. Flow Cytometry and Binding Studies

**Figure 1:**
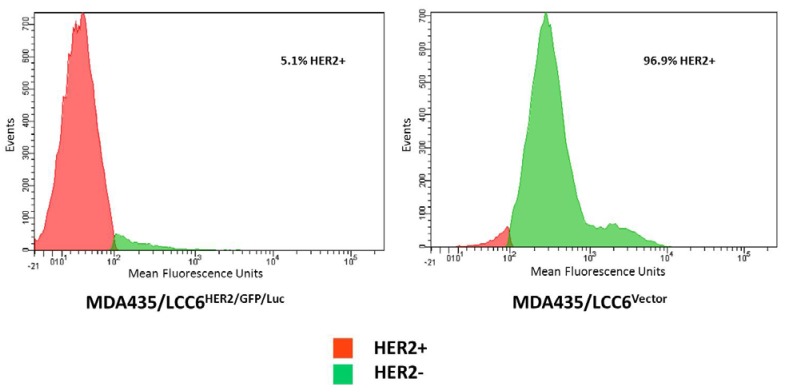
Flow cytometric analysis of HER2/neu expression. The MDA-MB-435-HER2-luc and MDA-MB-435 breast cancer cell lines were evaluated for HER2/neu expression. Trastuzumab was used as the primary antibody, and FITC-conjugated goat anti-human IgG was used as the secondary antibody. Data are shown as cell number on the ordinate access and HER2/neu intensity on the abscissa.

To evaluate the relative expression levels of the HER2 expression on each cell line, flow cytometry was performed. MDA435/LCC6^HER2/GFP/Luc^ (HER2+) cells demonstrated elevated levels of HER2 expression in comparison to MDA435/LCC6^Vector^ (HER2−) cells which demonstrated minimal expression (Figure 1).

**Figure 2:**
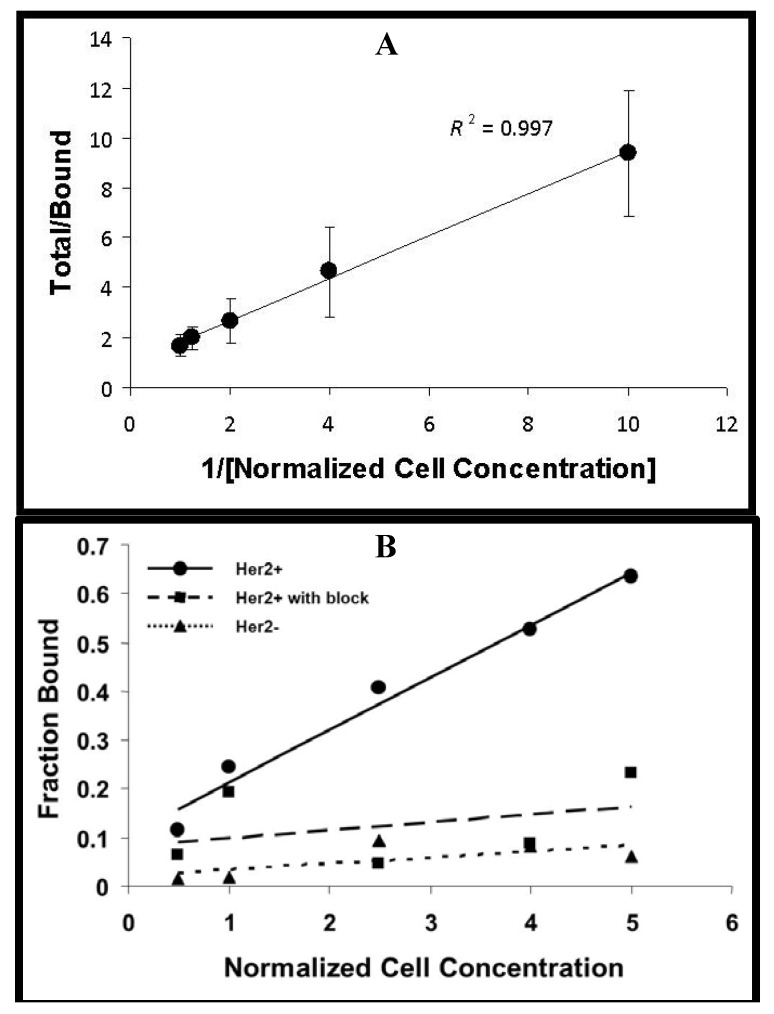
.Cell binding studies. (**A**) Plot of the (total/bound) activity *versus* (1/[normalized cell concentration]), used to calculate the immunoreactive fraction of ^89^Zr-Df-NCS-trastuzumab in MDA-MB-435-Her2-luc cells by extrapolation to the y-intercept; (**B**) Cell uptake curves of Fraction bound *versus* normalized cell concentration in MDA-MB-435-Her2-luc and MDA-MB-435-luc. A blocking dose of unlabeled trastuzumab inhibited the binding of ^89^Zr-radiolabeled trastuzumab.

The immunoreactive fraction of ^89^Zr-trastuzumabwas 95.6% (Figure 2A). The uptake of ^89^Zr-trastuzumab was 8.5-fold greater in the HER2+ cells compared to the HER2− cells (P < 0.01) (Figure 2B). Pre-incubation with excess unlabeled trastuzumab significantly inhibited the uptake of ^89^Zr-trastuzumab in HER2+ cells (Figure 2B).

### 3.3. Biodistribution Studies

**Figure 3:**
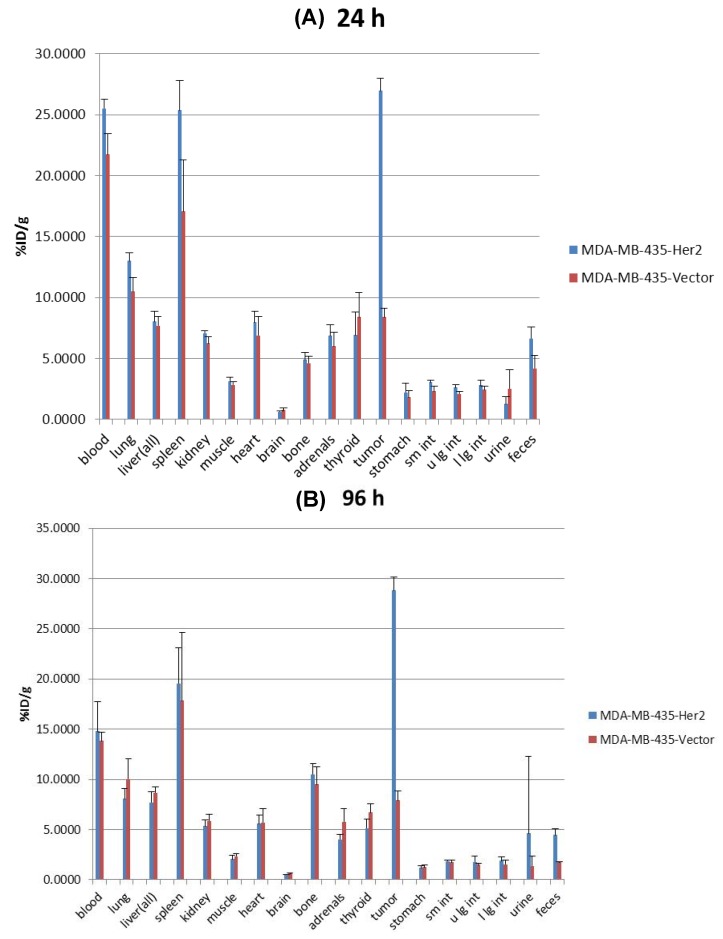
Biodistribution of ^89^Zr-radiolabeled trastuzumab in MDA-MB-435-Her2-luc and MDA-MB-435-luc orthotopic xenograft models at (**A**) 24 h and (**B**) 96 h post-injection. Data are expressed as percent injected dose per gram ± standard deviation, n = 5 for each time point.

Biodistribution studies with ^89^Zr-trastuzumab were performed in athymic nude mice bearing HER2+ or HER2− tumors. Uptake of ^89^Zr-trastuzumab in HER2+ tumors was elevated after 24 h (26.9 ± 1.04% ID/g) and continued to remain elevated at 96 h post-injection (28.83 ± 1.33% ID/g) resulting in tumor to muscle ratios (T/M) of 8.7 ± 1.2 and 14.5 ± 2.9, respectively. In contrast, ^89^Zr-trastuzumab uptake in HER2− tumors was significantly lower at 24 h (8.40 ± 0.72% ID/g) and 96 h (7.91 ± 0.96% ID/g) in comparison to HER2+ tumors (P < 0.001 at 24 and 96 h), resulting in T/M of 3.0 ± 0.4 and 3.4 ± 0.6, respectively. The circulating levels of ^89^Zr-trastuzumab in the blood declined from 23.6 ± 2.4% ID/g at 24 h to 14.3 ± 2.1% ID/g at 96 h (P < 0.001). Bone uptake increased from 4.8 ± 0.5%ID/g at 24 h to 10.0 ± 1.4% ID/g at 96 h. The uptake for each selected organ at 24 and 96 h is shown in Figure 3A,B, respectively.

### 3.4. Imaging Studies

**Figure 4:**
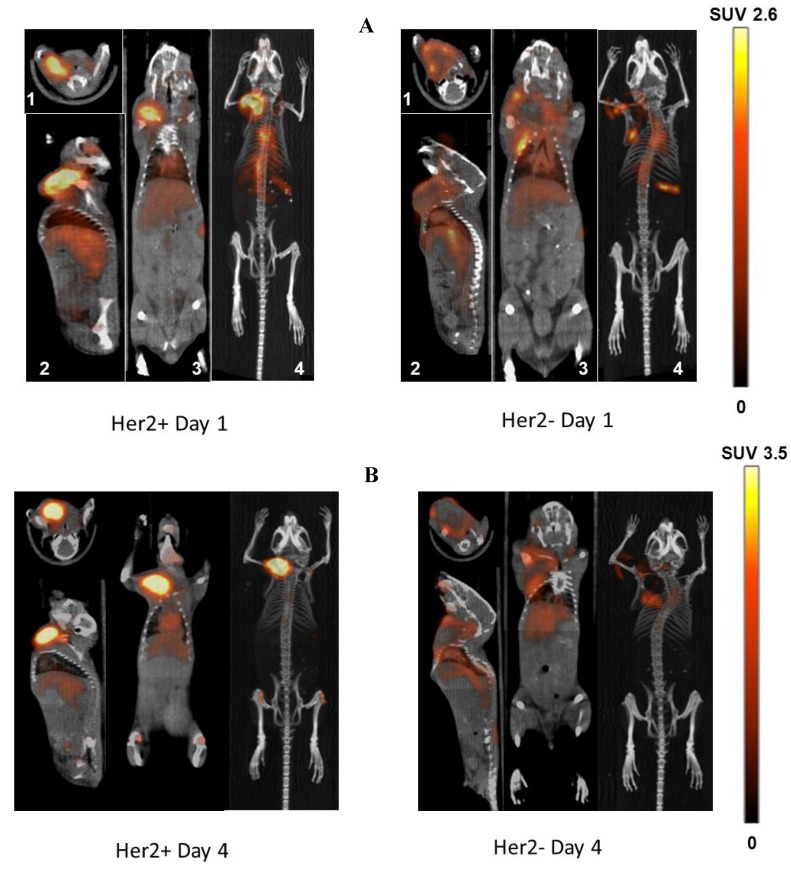
Representative microPET/CT images obtained at 24 and 96 h in (**A**) and (**B**) respectively. Tumors implanted in the right mammary fat pad of each mouse. The axial (1), sagittal (2), and coronal (3) slices at the center of the tumor are demonstrated along with the maximal intensity projection reconstruction (4). The scale expressed as standardized uptake value (SUV) is demonstrated at the far right.

MicroPET/CT imaging studies demonstrated high uptake of ^89^Zr-trastuzumab in HER2+ tumors when compared to HER2− tumors at 24 h and 96 h (Figure 4A,B, respectively). The uptake in HER2− tumors was minimally increased compared to background. The SUV_mean_ for ^89^Zr-trastuzumab for HER2+ and HER2− were 2.4 ± 0.3 and 1.4 ± 0.3 at 24 h (P = 0.003), and 3.1 ± 0.4 and 1.2 ± 0.1 at 96 h (P < 0.001), respectively. The level of ^89^Zr-trastuzumab in the blood significantly decreased from 24 to 96 h (SUV_mean_ 1.7 ± 0.2 and 1.2 ± 0.3, respectively) as evidenced by visualization of the decreased radioactivity in the blood pool in the heart and the inferior vena cava. Uptake in the liver (SUV_mean_ 0.82 ± 0.4) and kidney (SUV_mean_ 0.58 ± 0.3) were low compared to HER2+ tumors at 96 h.

**Figure 5:**
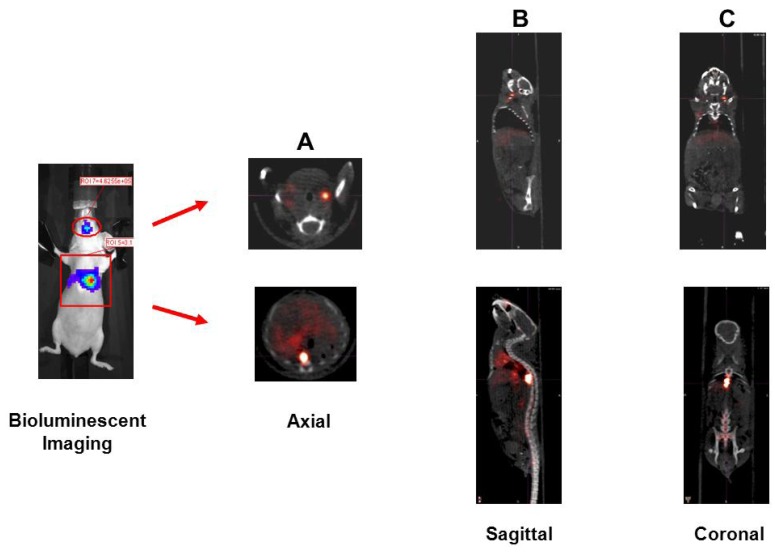
Detection of Her2+ metastatic lesions. 2 × 10^6^ MDA-MB-435-Her2-luc cells were administered via tail vein injection. Six weeks later, bioluminescent imaging (far right panel) demonstrated development of metastasis in the head and thoracic region. MicroPET/CT imaging using ^89^Zr-radiolabeled trastuzumab was able to localize these lesions to the L jaw and thoracic vetebral body. Shown are the axial (**A**), sagittal (**B**), and coronal (**C**) slices at the center of the tumor.

For the metastatic tumor model, bioluminescent imaging demonstrated the development of metastasis in the jaw (SUV_mean_ 2.6) and in the mid-thorax region (SUV_mean_ 2.9). These lesions were readily visualized by microPET/CT demonstrating the potential use of ^89^Zr-trastuzumab for characterizing HER2 expression in metastatic lesions (Figure 5).

### 3.5. Immunohistochemistry

**Figure 6:**
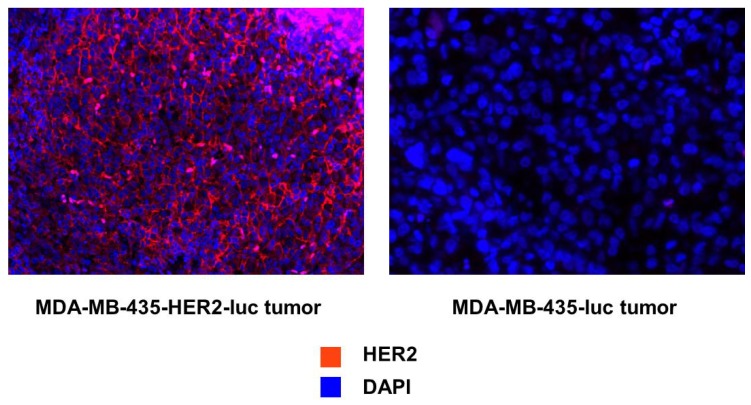
Immunofluorescent staining to evaluate the relative levels of HER2/neu expression for each tumor type. HER2/neu staining is demonstrated in red with a counterstain for the nucleus in blue.

To confirm that HER2 expression levels in tumors related to HER2 expression *in vitro*, immunofluorescence staining was performed with an anti-HER2 antibody. Images were acquired under the same conditions and displayed on the same scale to ensure that the relative brightness observed in images reflected differences in HER2 expression level. A high level of HER2 staining (red) was seen in the HER2+ tumors when compared with the HER2− tumors. DAPI (blue) was used as the counterstain for the nucleus (Figure 6).

### 3.6. Discussion

In the current study, elevated ^89^Zr-trastuzumab uptake was associated with the HER2 overexpression in breast cancer cells in both orthotopic mammary fat pad and metastatic tumor models. HER2+ tumors were clearly distinguished from HER2− tumors by microPET/CT imaging with this agent. Elevated ^89^Zr-trastuzumab uptake in HER2+ tumors in comparison to low normal tissue uptake resulted in high resolution images with excellent contrast between the tumor and normal tissues. The circulating blood levels of ^89^Zr-trastuzumab continued to decline over 4 days while the uptake in HER2+ tumors remained elevated.

Previous studies utilized a multi-step procedure with a succinylated derivative of desferrioxamine B for conjugation to trastuzumab. Vosjan *et al*. have reported a simple, efficient method for the radiolabeling of antibodies with ^89^Zr [35]. However, this procedure has not been evaluated for ^89^Zr-radiolabeling of trastuzumab. In the current study, the ^89^Zr-radiolabeling of trastuzumab was completed in approximately 3 h. The relative ease of the two-step procedure used in the current study makes it feasible for incorporation into good manufacturing practice grade production and for widespread clinical use.

Both an orthotopic mammary fat pad and metastatic model was utilized in the current study. ^89^Zr-trastuzumab produced with the simpler method was able to characterize HER2 expression in tumors from the orthopic and metastatic mouse models. While previous studies utilized heterotopic subcutaneous xenograft models for ovarian and breast cancer [24,39,40], the orthotopic model may be more clinically relevant by mimicking the tumor microenviroment and vasculature. Our results are in agreement with Dijkers *et al*., who reported an uptake value of ~33% ID/g in HER2/neu positive hindlimb tumors. In contrast, Holland *et al*. reported 2.5-fold higher uptake values (~73–86% ID/g) in subcutaneous shoulder tumors [39]. These differences may be explained by the different levels of HER2/neu expression in the varying cell lines used between the studies and also by differences in tumor perfusion due to the different locations of tumor growth.

The metastatic breast cancer model utilized in the current study was developed via tail vein injection of HER2+ cells. The development of tumors was initially monitored via bioluminescent imaging. This model is more of a measure of tumor invasiveness rather than metastasis as distant sites are not derived from cells migrating from a primary tumor. Despite this caveat, this model was suitable for evaluating the ability of ^89^Zr-trastuzumab to detect HER2 expression in distant sites of disease. The 3.3 day half-life of ^89^Zr is ideal for imaging full, intact antibodies (150 kDa) which require multiple days for equilibration as demonstrated in this study and previous studies [40]. Because ^89^Zr is relatively biologically inert, ^89^Zr-radiolabeled antibodies have low background uptake and improved stability in comparison to other radionuclides such as ^64^Cu, which is readily transchelated by liver superoxide dismutase resulting in elevated liver uptake, or some ^124^I-radiolabeled compounds which are may be dehalogenated and taken up by the thyroid. There is some residual bone uptake of ^89^Zr (5–10%) which may be attributed to a slow rate of intratumoral metabolism of ^89^Zr-trastuzumab leading to transmetalation of ^89^Zr^4+^ ions which are sequestered in the bone [39]. Further studies on the metabolism of ^89^Zr-trastuzumab are underway by our group and others.

^89^Zr-trastuzumab has significant clinical potential for the detection and characterization of lesions for HER2 status. Confirmation of HER2 status is necessary prior to initiation of trastuzumab therapy. Immunohistochemical staining and fluorescence-*in*-*situ* hybridization techniques from biopsy specimens taken at initial diagnosis are the current standards for determining HER2 expression [13]. However, tumor heterogeneity plays a significant role in breast cancer [41] and HER2 expression and gene amplification may vary across the primary tumor. Moreover, differences in the extent of HER2 expression between the primary tumor and across metastatic lesions have been reported [17,42]. As demonstrated in this study, PET imaging with ^89^Zr-trastuzumab provides a comprehensive view of the HER2 status not only within the primary tumor, but also across metastatic lesions. PET imaging with ^89^Zr-trastuzumab may assist in the stratification of patients by HER2 status for decision-making about trastuzumab therapy. With the increased costs of healthcare, a method to select patients that benefit from trastuzumab therapy, while avoiding the unnecessary treatment of patients with low HER2 expressing tumors could have an impact on cancer treatment.

Although beyond the scope of this study, PET with ^89^Zr-trastuzumab may improve the ability to detect lesions not previously detected on routine staging studies such as CT, MRI, or bone scan. In a feasibility study involving 14 patients with metastatic breast cancer, ^89^Zr-trastuzumab PET imaging provided complementary information to CT, MRI, and bone scan [28]. Of note, ^89^Zr-trastuzumab PET imaging was able to reveal previously undetected brain metastases [28]. Determination of the sensitivity and specificity of ^89^Zr-trastuzumab PET imaging and correlation with HER2 status in pathological human specimens will be challenging as it is unethical to subject patients to the discomfort of additional unnecessary biopsies.

Quantitative pre-therapy imaging studies with ^89^Zr-radiolabeled trastuzumab may also provide individualized pharmacokinetic and biodistribution information necessary to determine the optimal dose of trastuzumab for each patient. Scouting studies with ^89^Zr-radiolabeled trastuzumab may provide information regarding tumor uptake in comparison to critical normal organs allowing for maximal tumor targeting while minimizing normal tissue toxicity. Thus, ^89^Zr-trastuzumab imaging can provide this information in an efficient and safe manner, with fewer patients treated at a suboptimal dose.

## 4. Conclusions

The *p*-isothiocyanatobenzyl derivative of desferrioxamine B was utilized for radiolabeling of trastuzumab. Although beyond the scope of this study, this two-step procedure may be easily applied to preparation of ^89^Zr-radiolabeled trastuzumab for clinical use. Biodistribution and imaging studies demonstrated comparable results to those previously reported with the succinylated deravitave of desferrioxamine used in preparation of ^89^Zr-trastuzumab. ^89^Zr-trastuzumab PET imaging is a non-invasive method for characterizing HER2 expression in primary and distant lesions. This information may be useful in selecting patients that benefit most from trastuzumab therapy.
